# Increased spinal prodynorphin gene expression in reinflammation-associated hyperalgesia after neonatal inflammatory insult

**DOI:** 10.1186/1471-2202-11-139

**Published:** 2010-10-25

**Authors:** Jack Yu-Shih Lin, Yu-Che Cheng, Julia Yi-Ru Chen, Chih-Cheng Chien, Shih-Chang Lin, Yeong-Ray Wen, Tsung-Shan Tsou, Qing-Dong Ling

**Affiliations:** 1 Municipal Wan-Fang Hospital and Shung-Ho Hospital, Taipei Medical University, Taipei, Taiwan; 2 Graduate Institute of Systems Biology and Bioinformatics, National Central University, Chungli, Taiwan; 3 Cathay Medical Research Institute, Cathay General Hospital, Jian-Cheng Road, Sijhih City Taipei, Taiwan; 4 School of Medicine, Taipei Medical University, Taipei, Taiwan; 5 Department of Anesthesiology, Shin-Kong Wu Ho-Su Memorial Hospital, Wen Chang Road, Taipei, Taiwan; 6 Graduate Institute of Statistics, National Central University, Chungli, Taiwan

## Abstract

**Background:**

Neuroplasticity induced by neonatal inflammation is the consequence of a combination of activity-dependent changes in neurons. We investigated neuronal sensitivity to a noxious stimulus in a rat model of neonatal hind-paw peripheral inflammation and assessed changes in pain behaviour at the physiological and molecular levels after peripheral reinflammation in adulthood.

**Results:**

A decrease in paw withdrawal latency (PWL) after a heat stimulus was documented in rats that received inflammatory injections in their left hind paws on postnatal day one (P1) and a reinflammation stimulus at postnatal 6-8 weeks of age, compared with normal rats. An increase in the expression of the prodynorphin (*proDYN*) gene was noted after reinflammation in the spinal cord ipsilateral to the afferents of the neonatally treated hind paw. The involvement of the activation of extracellular signal-regulated kinases (ERK) in peripheral inflammatory pain hypersensitivity was evidenced evident by the increase in phospho-ERK (pERK) activity after reinflammation.

**Conclusions:**

Our results indicate that peripheral inflammation in neonates can permanently alter the pain processing pathway during the subsequent sensory stimulation of the region. Elucidation of the mechanism underlying the developing pain circuitry will provide new insights into the understanding of the early pain behaviours and the subsequent adaptation to pain.

## Background

Since the clinical demonstration of varied hormonal and metabolic responses in infants undergoing surgery, which were attenuated by general anaesthesia, clinicians have suggested that marked nociceptive activity in premature or full-term neonates constitutes a physiological, and perhaps even a psychological, form of stress[1]. An increased focus on the neurobiology of developing pain pathways attests the awareness of the importance of pain in infancy[2]. Previous studies demonstrated clearly that peripheral inflammation experienced during the neonatal period has long-standing consequences on spinal nociceptive neuronal circuits[3,4]. In our recent studies, we have demonstrated that, during the process of neonatal neuronal development of the primary afferents following neonatal peripheral inflammation, there is not only a dynamic change in the pattern and distribution of calcitonin gene-related peptides (CGRP) containing terminals in various regions of the dorsal horn,[5] but also a molecular change in neurotrophic factors, particularly the nerve growth factor (NGF), and brain-derived neurotrophic factor (BDNF) [6]. Furthermore, as behavioural,[7] electrophysiological [8] and immunohistochemistry [9] studies have shown, these changes induce a subsequent hypersensitization in response to later sensory stimulation and noxious stimulation.

Thus, it is well established that the central nervous system is active during prenatal development, and that detrimental and developmental changes resulting from inflammatory insults lead to central excitability alterations, which modify later pain-stimulated behaviour patterns[10,11]. However, little is known about the mechanism that underlies, and the developmental nature of, these alterations. In this study, we assessed the variation in the levels of the proDYN mRNA during the long-term modulation of nociceptive neuronal circuits after neonatal Complete Freund' Adjuvant (CFA) -induced peripheral inflammation.

Dynorphin is a class of endogenous opioid peptides that are produced by many different populations of neurons in the hypothalamus, hippocampus and spinal cord[12]. Although this peptide is classified as an endogenous opioid peptide that binds to the opioid kappa receptors, numerous studies indicate that much of the pharmacology of this peptide is dependent on its interaction with NMDA receptors, rather than with opioid receptors[13]. Several groups reported that the increase in spinal dynorphin expression after peripheral noxious stimulation [14-16] was mediated by the mitogen-activated protein kinases (MAPK)/extracellular signal-regulated kinases (ERK) pathway [17] via a positive-feedback mechanism, which results in neuropathic and other chronic (e.g., inflammatory) pain states[18,19]. Moreover, the intrathecal administration of dynorphin induces behavioural signs of hyperalgesia similar to those observed in central hypersensitization induced by peripheral inflammation or nerve-injury-induced pain[20,21]. These experiments support the previous hypothesis that pathological or upregulated levels of spinal dynorphin play a pro-nociceptive role by maintaining "central sensitization" in the post-nerve injury state[22,23]. In this study, we examined the role of the MAPK/ERK pathway in the upregulation of dynorphin in the reinflammation-associated hyperalgesia observed in adult rats that experienced neonatal inflammatory insults. Behaviour profiles, gene expression and *in situ *hybridization studies were performed to substantiate our postulation.

## Results

### Behavioural responses to noxious heat stimuli at different time points after reinflammation

**Figure 1:**
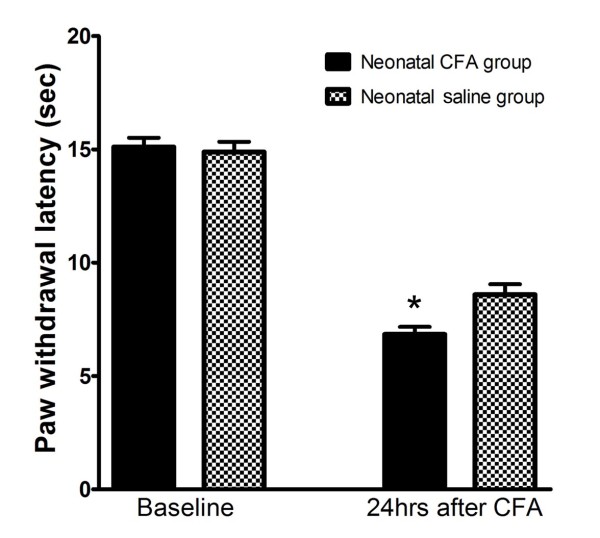
**Behavioural analysis using thermal tests after reinflammation**. The PWL to a noxious thermal stimulus at baseline and at 24 h after CFA reinflammation was compared between the neonatal CFA group and the neonatal saline group. The results revealed an absence of differences in the baseline PWL to a noxious thermal stimulus between the neonatal CFA group and the neonatal saline group. However, 24 h after reinflammation via CFA injection into the left hind paw, the PWL in the neonatal CFA group was significantly decreased compared with that observed in the neonatal saline group (* P < 0.05).

PWL was evaluated in the neonatal CFA and saline groups 24 h after reinflammation via CFA injection into the left hind paw at postnatal age of 6-8 weeks. No difference in the baseline PWL was found in the neonatal CFA group (n = 20) compared with the neonatal saline group (n = 20) (Figure 1). The mean PWL was 15.10 ± 0.41 s for the left hind paw in the neonatal CFA group and 14.88 ± 0.46 s for the left hind paw in the neonatal saline group. Twenty-four hours after reinflammation via CFA injection in the left hind paw, the neonatal CFA group showed a significant decrease in PWL in the left hind paws compared with the neonatal saline-injected left hind paws. The mean PWL was 6.84 ± 0.33 s and 8.59 ± 0.46 s, respectively.

### Comparison of proDYN mRNA expression using real-time RT-PCR

**Figure 2:**
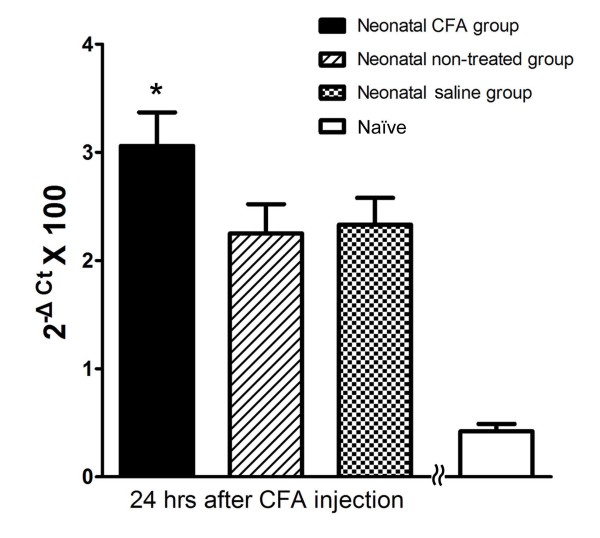
**Determination of proDYN mRNA expression levels in the spinal cord after reinflammation using real-time RT-PCR**. A significant increase in proDYN mRNA expression was found in the neonatal CFA group compared with the neonatal non-treated and neonatal saline groups 24 h after reinflammation with CFA (* P < 0.05). No significant differences in proDYN expression levels was noted between the neonatal non-treated group and the neonatal saline group. Little or no proDYN expression was noted in the naïve group, which did not receive any CFA injection during the neonatal and the adult time.

Real-time RT-PCR was performed to compare the relative proDYN mRNA expression levels in the left side of the spinal cord (L4-5) in each group 24 hours after CFA injection into the left hind paw at postnatal age of 6-8 weeks, with standardization to the naive rats (Figure 2). The Ct relative quantification method was used with the level of the proDYN mRNA expression presented as the value of 2^-ΔCt^, as described in the method section. A significant increase in proDYN mRNA expression was detected in the neonatal CFA group (n = 10) compared with its expression levels in the neonatal saline group (n = 10), the neonatal non-treated group (n = 10) and the naive group (n = 10). The 2-ΔCt × 100 values were 3.06 ± 0.31, 2.25 ± 0.27 and 2.33 ± 0.25 and 0.24 ± 0.07, respectively. Since the naïve group did not receive any CFA injection during the neonatal and the adult period, little or no proDYN mRNA expression was noted. In contrast, no significant difference in proDYN mRNA expression was found between the neonatal non-treated group and the neonatal saline group.

### In situ hybridization labelling of proDYN mRNA in the lumbar dorsal horn

**Figure 3:**
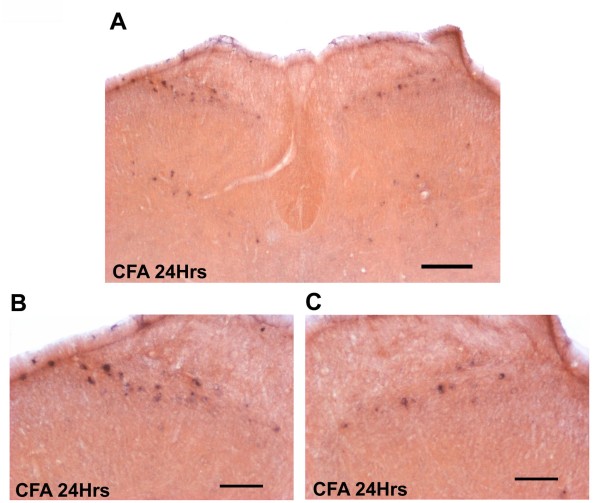
**In situ hybridization study showing an upregulation of the proDYN mRNA in the dorsal horns after reinflammation**. A low-magnification image shows neurons expressing spinal proDYN mRNA in the superficial and deeper laminae of the dorsal horn 24 h after reinflammation in the CFA group (A). A high-magnification image of Figure 3A shows a comparable increase in proDYN mRNA-positive neurons in laminae I-II of the dorsal horn ipsilateral (B) side that experienced neonatal peripheral inflammation compared with the contralateral side, which did not receive neonatal peripheral CFA insults (C). Scale bars: a, 200 μm; b and c, 100 μm

**Figure 4:**
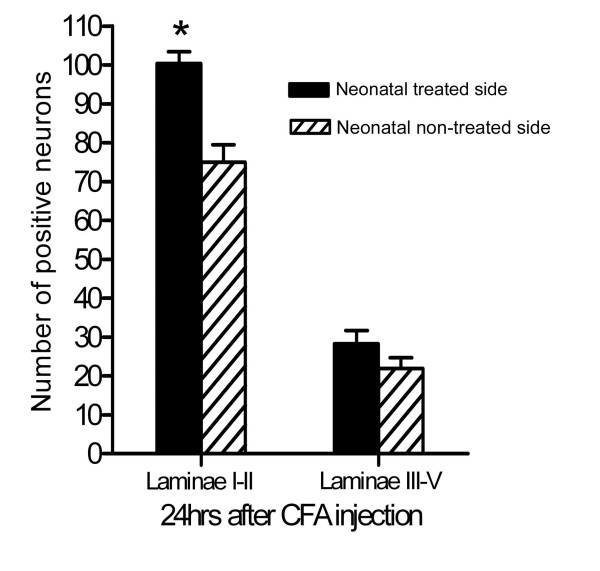
**Quantification of proDYN mRNA-positive neurons using in situ hybridization in laminae I-II and laminae III-V of the dorsal horn 24 h after reinflammation with CFA injection into bilateral hind paws at postnatal time 6-8 weeks in the neonatal CFA group**. A significant increase in the number of proDYN mRNA-positive neurons was found in laminae I-II of the left side that received a neonatal peripheral CFA insults compared to the right dorsal horn, which did not receive neonatal CFA insults (* P < 0.05). There was also an increase in the number of proDYN mRNA-positive neurons in laminae III-V of the neonatal CFA-treated side compared with the contralateral side; however, this difference was not significant.

In situ hybridization studies were performed to determine the levels of expression and the localization of the proDYN mRNAs in the dorsal horn of rats in the neonatal CFA group. Twenty-four hours after reinflammation with CFA in both hind paws of the neonatal CFA group at postnatal age of 6-8 weeks, there was a significant increase in the number of cells that expressed proDYN mRNA in the left dorsal horn, ipsilateral to the afferents of the neonatally CFA-treated left hind paw, compared to the right dorsal horn. The proDYN gene is primarily expressed in the superficial laminae (laminae I and II) and the deeper laminae (laminae III and IV) of the dorsal horn (Figure 3A). A higher magnification of the superficial laminae revealed that the proDYN mRNA-positive cells were mainly distributed in laminae I and II, with a higher intensity of labelling in the left dorsal horn (Figure 3B) compared with the right dorsal horn (Figure 3C). The comparison of the number of proDYN-positive cells between the left and right superficial dorsal horns revealed that the mean spinal cord laminae I- and II-positive ganglia on the left side was 100.33 ± 5.17, vs. 75.00 ± 7.77 on the right side. The difference was significant (P < 0.05). The mean L4/L5 laminae III to V positive cells on the left side was 28.33 ± 5.84, vs. 22.00 ± 4.62 on the right side. The difference was not significant (Figure 4).

### pERK activation after CFA-induced peripheral inflammation

**Figure 5:**
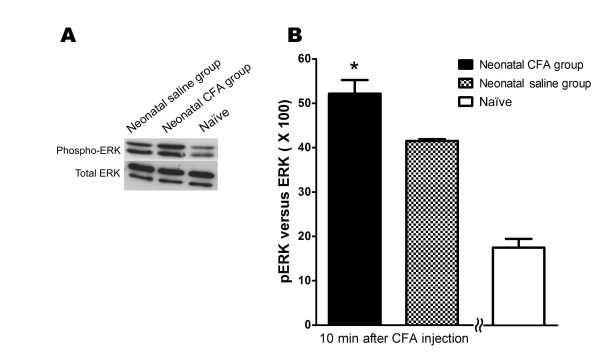
**Western blot analysis of pERK activation in spinal cords subjected to CFA-induced peripheral reinflammation**. Western blots of spinal cord tissues from animals in the neonatal CFA group, neonatal saline group and the naive group revealed varied immunoreactivity against different isomers of pERK 10 min after reinflammation (A). The intensity of the total ERK was the same in the three groups. A significant increase in pERK activity was found in the spinal cord tissues of animals in the neonatal CFA group compared with the neonatal saline group and the naive group (* P < 0.05) (B).

To investigate whether the cellular MAPK/ERK signal pathway was involved in spinal pain signal transduction, the levels of pERK and total ERK proteins extracted from the spinal cord of animals in the neonatal CFA and saline groups 10 min after peripheral reinflammation were examined using Western blotting. Staining of the different isomers of the pERK protein revealed an increase in density in the neonatal CFA group (n = 6) compared with that observed in the neonatal saline (n = 6) and the naive groups (n = 6) (Figure 5A). The intensity of the total ERK proteins was the same in all groups. The Western blots supports Figure 5B, which shows that, 10 min after reinflammation with CFA at postnatal age of 6-8 weeks, the ratio of pERK to total ERK (pERK/ERK) in the neonatal CFA group was significantly higher compared with the ratio observed in the neonatal saline group and in the naïve group (P < 0.05). This suggests an increased pERK protein concentration in the neonatal CFA group.

## Discussion

Advances in medical technology led to a substantial improvement in the chances of survival of medically compromised newborns via the development of therapies, which often inflict tissue injury and pain. The existence of background pain in these children not only influences their acute pain experience but often also leads to a significantly lower pain threshold compared with their healthy peers, which is independent of various psychological factors[24]. Studies showed that early injury in the first postnatal weeks leads to the development of neural circuits that permanently alter pain perception[25]. Furthermore, it was also demonstrated that short-lasting inflammatory insults during the neonatal period generate hypoalgesia in adults[4,7,26]. This is compatible with the present behaviour studies, which revealed a generalized reduction in baseline sensitivity in response to a noxious heat stimulus. In contrast, neonatal injury can also have the opposite effect. Long-term hyperalgesia, although masked at the segmental level,[27-29] was uncovered after a much greater stimulus (such as reinflammation) was applied. This reinflammation-associated hyperalgesia reflected an inflammatory challenge, which strengthens the nociceptive signal transduction pathway [8,9] and leads to a reduction in hypoalgesia[30]. Although the signalling system that underlies the adult hyperalgesia observed after neonatal peripheral inflammation is poorly understood, it is now known that neural plasticity is fully functional at birth and the somatosensory nervous system, though immature, is fully capable of transmitting sensory information[25,31]. In the present study, a decrease in PWL was found 24 h after CFA-induced reinflammation in the neonatal CFA group compared with the neonatal saline group. This observation was consistent with our previous results [3,30] and with results from other studies[4,26]. These studies all point to the conclusion that increased pain sensitivity in adults may develop in rats that experienced peripheral inflammatory insults during the neonatal period.

Numerous factors have been implicated in hyperalgesia as outcomes of early peripheral noxious experience, including substance P, the NMDA receptor, pERK, dynorphin, and c-fos[17,32-36]. However, studies that looked into the mechanism that underlies the hyperalgesia induced by reinflammation in rats that received neonatal noxious stimuli are scarce. In the present study, the assessment of the expression of the proDYN gene using quantitative real-time RT-PCR revealed the upregulation of this gene after reinflammation in the spinal cord in the neonatal CFA group compared with the neonatal saline group, 24 hours after reinflammation via CFA injection. The in situ hybridization analysis showed that this increased expression of proDYN after reinflammation was primarily localized to the superficial laminae I and II of the dorsal horn ipsilateral to the site of the neonatal insult, in the area where nociceptive afferent projections, such as A-delta and C fibres, terminate. No significant difference was noted in the expression of proDYN in the neck of the dorsal horn (laminae V and VI) between the two sides of the spinal cord of the neonatal CFA group after the bilateral induction of reinflammation.

ProDYN, which has both nociceptive and anti-nociceptive properties,[23] plays an important role in promoting and maintaining neuropathic pain. Although dynorphin at submicromolar concentrations may activate opioid receptors to reduce calcium influx, thus leading to hypoalgesia,[21] at higher concentrations (10-100 μM) dynorphin is pro-nociceptive in some pathological pain states via interaction with the NMDA receptor, which leads to allodynia[17,20]. In our real-time RT-PCR experiments, we observed a significant increase in proDYN expression after reinflammation in adult rats that experienced inflammatory insults during the neonatal period (neonatal CFA group) compared with rats that received a saline injection during the neonatal period (neonatal saline group). Concomitantly, the neonatal CFA group also exhibited behaviours of hyperalgesia, which manifested via a significant decrease in PWL compared with the neonatal saline group. This peripheral-inflammation-associated hyperalgesia was previously discussed in a study by Dubner and Ruda[15]. These authors found that an increase in neuronal activity in response to tissue injury may lead to changes in gene expression and prolonged changes in the nervous system, which may contribute to hyperalgesia. This increase was most evident 24 h after the inflammation insult. This activity-dependent plasticity may involve nociceptive processing neuropeptides, such as dynorphin, substance P and the calcitonin gene-related peptide. In fact, previous papers have shown that spinal dynorphin may promote pain, in part, by enhancing the evoked release of excitatory transmitters such as CGRP from primary afferents in the dorsal root ganglia[37]. Neurons exhibiting proDYN upregulation in the superficial and deep laminae of dorsal horn were projection neurons that convey nociceptive information. Our previous studies revealed that a neonatal inflammatory stimulus resulted in an increase in the expression of the neurotrophin receptor gene in dorsal root ganglia during the early postnatal period[6]. Release of growth factors, such as NGF, has been implicated with an increase in the terminal density of nociceptors both in the spinal dorsal horn [5] and in the injured region, which alters the development of the nociceptors[38,39]. Similarly, the upregulation of proDYN secondary to a peripheral inflammatory insult during the neonatal period can also be associated with the increase in the density of neuronal terminals, which may occur during the period of the neonatal insults.

In this study, we also investigated the role of the MAPK/ERK pathway in the modulation of nociceptive neuronal circuits in rats that received CFA-induced peripheral insults during the neonatal period. We detected the upregulation of pERK in the spinal cord of rats in the neonatal CFA group compared with the neonatal saline group, after reinflammation. ERK plays a pivotal role in functional nociceptive plasticity, which in turn contributes to altered sensibility[40]. Previous studies pointed out that ERK activation is likely to regulate the expression of proDYN via transactivators, such as pCREB and c-fos[41,42]. The link between the ERK activation and proDYN expression was investigated in a study by Woolf et al. in which they used a MEK Inhibitor U0126, which is a chemically synthesized organic compound that inhibits the kinase activity of MAP kinase, to block ERK activation and subsequently found a decrease in the CFA-induced proDYN mRNA expression[17]. Our results were compatible with the findings of other studies, in that an increased pERK level was associated with an increase in the expression of the proDYN mRNA, which may contribute to the development of reinflammation-induced pain hypersensitivity (decreased PWL) after neonatal peripheral inflammation.

## Conclusion

This study explored the molecular mechanisms that underlie adult pain hypersensitivity after neonatal peripheral inflammatory insults. The spinal upregulation of endogenous proDYN observed in our model may participate in the development of reinflammation-associated pain hypersensitivity in adults after neonatal peripheral inflammatory insults. The study of the development of the nociceptive showed that pain in newborns involves functional signalling pathways that are not found in the mature nervous system of adults[31]. The results of our studies suggest that peripheral inflammation in neonates could result in lasting alterations in nociceptive pathways. We postulate that the activation of dynorphin via the MAPK/ERK pathway contributes to the reinflammation-associated hyperalgesia observed in adult rats that experienced neonatal peripheral inflammatory insults.

## Methods

### Animals and inducement of inflammation

Timed-pregnant Sprague Dawley rats were monitored to determine the time of birth of litters. One hundred neonatal rats were separated into four groups. The first group included rats that received Complete Freund' Adjuvant (CFA) injections into their left hind paws both on postnatal day 1 and again at postnatal age of 6-8 weeks (neonatal CFA group; n = 34). The second group received a saline injection into the left hind paws on postnatal day 1 and CFA injection at postnatal age of 6-8 weeks (neonatal saline group; n = 34). The third group did not receive any kind of injection into their left hind paws on postnatal day 1, but received CFA injection at postnatal age of 6-8 weeks (neonatal no-treatment group; n = 22). The last group of rats did not receive any injection on postnatal day 1 or at postnatal age of 6-8 weeks (naïve; n = 10). Inducement of neonatal inflammation and adult reinflammation were performed as described in Ruda et al[30]. For the neonatal CFA group, each male rat pup received a single, unilateral subcutaneous injection of CFA into the left hind paw (CFA:saline, 2:1; 25 μl) (Sigma Chemical Co., St. Louis, MO) on postnatal day 1. The same volume of saline was injected into the left hind paw of animals in the neonatal saline group, on the same day. The animals were allowed to mature to adulthood without further manipulation. To induce reinflammation in adult rats in the neonatal CFA group, the neonatal saline group, and the neonatal no-treatment group at the postnatal age of 6-8 weeks (P42-56), 200 μl of CFA (CFA:saline,1:1) were injected unilaterally into the plantar surface of the hind paws, to perform behaviour assessment of these animals, as well as the gene expression quantification and Western blotting experiments. For the in situ hybridization experiments, the CFA-induced reinflammation was applied bilaterally in the neonatal CFA group at postnatal age of 6-8 weeks, to compare the number of proDYN-positive nuclei between the two sides of the spinal column. All procedures used in this study were approved by the Animal Research Facility of Cathay Medical Research Institute, Cathay General Hospital, and followed the guidelines for the treatment of animals of the International Association for the Study of Pain[43].

### Behavioural assessments

The PWL to a noxious radiant heat stimulus was determined as described in Hargreaves et al[44]. At 8-10 weeks of age, the baseline PWL of the left hind paw to a radiant heat source was determined in rats in the neonatal CFA group and in the neonatal saline group. Heat hyperalgesia was tested 24 hours after the injection of CFA into the left hind paw and was measured four times at intervals of 5 min. PWL was calculated by combining and averaging the mean latencies of three stimulus presentations to each hind paw, excluding the first familiarization trial.

### Tissue collection

Rats in the neonatal CFA group and neonatal saline group were euthanized after CFA injection. For the quantification of mRNA expression levels, animals from each time point of the behavioural experiments were euthanized via intraperitoneal injection of an overdose of sodium pentobarbital (50 mg/kg). The L4 and L5 dorsal root ganglia (DRG) were exposed and their roots were traced up to the entry points on the spinal cord using a surgical microscope. The lumbar spinal cord containing the L4-5 segments was removed and the tissue was sectioned along the midline into the left (i.e., projecting side of neonatal CFA-treated animals) and right sides. Tissues were frozen at -80°C until the isolation of RNA. For the in situ hybridization experiments, rats were deeply anesthetized with pentobarbital and perfused transcardially with saline, which was followed by incubation in 4% paraformaldehyde in 0.1 M phosphate buffer (pH 7.4). The L4-5 spinal cord segments were removed and postfixed for 2-4 h before transferring to a 30% sucrose/PBS solution and incubation overnight at 4°C.

### Isolation of RNA and real-time RT-PCR quantification

Total RNA was isolated using the 3-Zol reagent (MDBio, Inc., Taipei, Taiwan) method and the RNA samples were treated with DNase I (Ambion, TX, USA) to remove traces of genomic DNA. To ensure optimal DNase I activity, the buffer conditions in the RNA solution were adjusted accordingly. RNA absorbance was measured at 260 nm using a spectrophotometer to obtain a yield in microgram per microlitre (μg/μl). TaqMan Gene expression assays (Applied Biosystems, NJ, USA) were used in a two-step RT-PCR process. First-strand cDNA was synthesized from 2 μg of total RNA using SuperScriptTM (Invitrogen, CA, USA) in 10 μl of total reaction solution. Real-time PCR reactions were then performed using an ABI PRISM 7300 Sequence Detection System (Applied Biosystems, NJ, USA). The sequence of the published proDYN cDNA was obtained from GenBank, of the National Center for Biotechnology Information (GenBank: NM019374). The actual sequences of the upstream and downstream PCR primers and of the probe oligonucleotide for proDYN were as follows: upstream primer, 5'-CGGCCATCCTATCACCTGA-3'; downstream primer, 5'-CCTTCCTGCGTGCTGCTT-3'; probe oligonucleotide, 5'-(6-FAM)CAGCCAGAAGCCTGCCAGCGA-3', where 6-FAM represents 6-carboxyfluorescein. The β-actin housekeeping gene was similarly amplified using TaqMan Rodent Control Reagents. For gene quantification, 2 μl of RT reaction were combined with 1 × TaqMan Universal Master Mix (Applied Biosystems, NJ, USA). The reactions were then thermally cycled for 10 min at 95°C, followed by 40 cycles of denaturation (15 s at 95°C) and annealing/extension for 60 s at 60°C. Data were then collected via instrument spectral compensations using the ABI PRISM Sequence Detection Software, version 1.6.3 (Applied Biosystems, NJ, USA), and analysed using the threshold cycle (Ct) relative quantification method (the 2^-ΔCt ^method). The Ct indicates the fractional cycle number at which the amount of amplified target reaches a fixed threshold. This method is used to determine the effect of the experimental treatment on the expression of a candidate gene against the internal control gene; in our study, the proDYN gene and the β-actin gene, respectively. For each of the mRNA sample, the value of ΔCt was calculated and normalized by taking the average Ct value for the proDYN gene minus the average Ct value for the internal control gene β-actin in the same RNA preparation. The value of the normalized proDYN gene expression against the internal control gene β-actin gene expression was then indicated by the value of 2^-ΔCt ^× 100 [45].

### Protein extraction and Western blot analysis

Frozen tissues were ground into a powder and resuspended in RIPA buffer (50 mM Tris-HCl, pH7.5, 150 mM NaCl, 10 mM EDTA, 1% NP-40 and 0.1% SDS) with the Protease Inhibitor Cocktail (Roche, Basel, Switzerland) and the Phosphatase Inhibitor Cocktails I and II (Sigma St. Louis, MO, USA). The suspended solution was sonicated on ice for 5 min. The debris was removed by centrifugation and the supernatant was used in subsequent experiments. Protein concentration was determined using the BCA protein assay kit (Pierce, Rockford, IL, USA) and bovine serum albumin was used as the standard. The samples were then loaded onto a 10% SDS-PAGE gel and subsequently transferred onto a PVDF membrane. Prior to sample application, each PVDF membrane (Perkin-Elmer, Boston, MA, USA) was sequentially preincubated with methanol and a buffer containing 48 mM Tris-HCl, 40 mM glycine, 0.0375% SDS and 20% methanol (pH 9.2). After the electrotransfer of proteins, the membranes were first incubated in TBST buffer (10 mM Tris-HCl pH 7.5, 150 mM NaCl, 0.05% Tween-20) containing 5% BSA (Sigma St. Louis, MO, USA) for 1 h at room temperature and were then probed with anti-ERK antibodies (Cell Signaling, Danvers, MA, USA) at a concentration of 1:1000 in TBST with 5% BSA for 1 h, or with anti-pERK antibodies (also termed anti-pMAPK) (Cell Signaling, Danvers, MA, USA) at a concentration of 1:200 in TBST with 5% BSA overnight. The membranes were washed with TBST four times and were then incubated with a horseradish peroxidase-conjugated goat anti-rabbit secondary antibody (Millipore, Billerica, MA, USA) at a concentration of 1:1000 in TBS for 1 h. The immunopositive proteins were detected using the ECL reagent (Pierce) and the chemiluminescence was visualized using Biomax MR film (Kodak, Rochester, NY, USA). The intensity of each band was quantified using the LabWorks software (UVP Bioimaging Systems, Upland, CA, USA).

### PCR amplification of rat proDYN cDNA fragments

Sets of specific primers were designed to amplify unique DNA fragments corresponding to regions of the rat proDYN gene using RT-PCR. The primer sequences for proDYN were as follows: 5'-GAGGACTTGAGAAAACAGGCC-3' and 5'-GGTATTGGGGTTCTCCTGGGA-3'. RT-PCR was implemented as follows. Total RNA (100 ng) was denatured at 70°C for 5 min and reverse transcribed into first-strand cDNA by priming with an oligo(dT) primer. PCR was performed using the Advantage PCR System (Clontech, CA, USA) with the following program cycles: one cycle (2 min at 95°C); 35 cycles (15 s at 94°C, 15 s at 50°C and 1 min at 68°C); and one cycle (10 min at 72°C). PCR products were resolved by electrophoresis on 1% agarose gels and the *proDYN *cDNA fragment (450 bp) was extracted using the QIAquick^® ^kit (QIAGEN, MD, USA).

#### In situ hybridization

To evaluate the expression of the proDYN gene, in situ hybridization was performed in the spinal cord of rats that received neonatal CFA treatment. Animals were euthanized 24 h after reinflammation with a bilateral injection of CFA at postnatal week 6-8. The L4-5 spinal cord segments were removed and sectioned on a cryostat at a thickness of 20 μm. The antisense RNA probe and the corresponding sense control probe were labelled via *in vitro *transcription using linearized DNA templates for proDYN and digoxigenin (DIG) labelling mixture (Roche, Mannheim, Germany) for 2 h at 37°C. *In situ *hybridization was processed as described previously[17]. Tissue sections were air dried for 2 h, fixed in 4% paraformaldehyde for 15 min and acetylated in acetic anhydride (0.25%) for 10 min. Sections were pre-hybridized for 2 h at room temperature and were then incubated in hybridization buffer overnight at 60°C. Sections were then washed in decreasing concentrations of SSC (2 ×, 1 ×, and 0.2 ×) for 2 h, blocked with 2% goat serum for 1 h and incubated overnight at 4°C with an alkaline-phosphatase-conjugated anti-DIG antibody (1:500; Roche, Penzberg, Germany). Finally, sections were visualized after being incubated in 75 μg/ml nitroblue-tetrazoliumchloride, 50 μg/ml 5-bromo-4-chloro-3-indolyl-phosphate, and 0.24 mg/ml levamisole for 6-8 h.

### Quantification and statistical analyses

Statistical evaluation was performed using GraphPad Prism 5.0 for Windows. All data are presented as the mean ± standard error (S.E.M.). The time course of PWL to thermal stimulation and the ΔCt of real-time RT-PCR were analysed in the radiant heat tests and gene expression experiments, respectively. Differences in the density of specific bands between groups were compared in the Western blot experiments. Three neonatal CFA rats were euthanized for the in situ hybridization experiment. Six nonadjacent sections from the L4-5 lumbar spinal cord of each of the three neonatal CFA rats were randomly selected and the number of mRNA-positive neuronal profiles in the superficial laminae and deep laminae of the dorsal horn of each section were counted blindly by an observer. Statistical comparisons of the values observed in response to reinflammation via CFA injection at different time points between the neonatal CFA group and the neonatal saline group were performed via analysis of variance (ANOVA) using the treatments as factors, followed by Tukey's post hoc comparisons among the treatment groups. Differences were considered significant at P < 0.05.

## Abbreviations

PWL: paw withdrawal latency; proDYN: prodynorphin; ERK: extracellular signal-regulated kinase; MAPK: Mitogen-activated protein kinase; CGRP: calcitonin gene-related peptide; NGF: nerve growth factor; BDNF: brain-derived neurotrophic factor; CFA: Complete Freund' Adjuvant; PBS: phosphate buffered saline; TBS: tris buffered saline; ANOVA: analysis of variance

## Authors' contributions

JYL, YCC and QDL planned, designed and conducted experiments. JYL, QDL wrote the manuscript. JYC, CCC, SCL, YRW and TST contributed some of interpretation in data analysis and revise aspects of the manuscript. All authors have read and approved the final manuscript.
